# Comment on “Ensuring the Efficacy and Safety of Approved Medications ”

**DOI:** 10.1007/s13181-024-01005-0

**Published:** 2024-05-02

**Authors:** J. Oppenheimer, Thomas B. Casale, Sarina Tanimoto

**Affiliations:** 1 UMDNJ Rutgers University School of Medicine, Newark, New Jersey USA; 2 Morsani College of Medicine, University of South Florida, Tampa, FL USA; 3 ARS Pharmaceuticals, Inc., 11682 El Camino Real, Suite 120, 92130 San Diego, California USA

**Keywords:** Epinephrine, Auto-injector, Intranasal, Anaphylaxis

Dear Editors,

The article by Mazer-Amirshahi et al. [1] discusses the approval process and data regarding intranasal epinephrine spray, *neffy* (ARS Pharmaceuticals, California) being developed as a treatment for severe allergic reactions including anaphylaxis.

We would like to correct the claim that the application submitted to the FDA was an Abbreviated New Drug Application as it was filed as New Drug Applications (NDA) under Sect. 505(b)(2) of the Federal Food, Drug, and Cosmetic Act. Approval of a change in route of administration for a known drug is routinely supported by pharmacokinetic data alone, with a long-standing precedence of approving 505(b)(2) NDA without efficacy studies. Where a drug’s mechanism of action (MOA) relies on systemic exposure, comparable pharmacokinetics is usually accepted given the route of administration should not impact the clinical outcomes. In neffy’s® case, the FDA also requested pharmacodynamic measures, based upon well-established MOA [2–3]. Additionally, the FDA required self-administration [4], repeat dose [2], and nasal allergen challenge (NAC) studies. The FDA Pulmonary Allergy Drug Advisory Committee (PADAC) reviewed the data and recommended neffy’s approval for pediatric patients with a vote of 17 to 5 [5].

ARS and the FDA agreed on a strategy to establish a scientific bridge between epinephrine injections and neffy, as conducting a randomized controlled study in patients experiencing anaphylaxis is deemed unethical or impractical [6–11]. ARS received feedback from the FDA indicating that Oral Food Challenge (OFC) or model anaphylaxis-type study would not offer additional valuable insights beyond the pharmacokinetic/pharmacodynamic studies agreed as part of the development program. The aim of these studies was to demonstrate pharmacokinetic/pharmacodynamic profile within the range or similar to that of approved injection products, which has already been demonstrated.

Since the PADAC meeting, ARS has completed additional clinical trials, including twice the dose after NAC, resulting in a pharmacokinetic/pharmacodynamic profile greater than or similar to intramuscular injection as well as a study in pediatric patients experiencing anaphylaxis symptoms following OFC [5], demonstrating a 100% response to a single dose within 15 min. To further study the clinical benefits of a common symptom of anaphylaxis, urticaria, patients with chronic urticaria received neffy® and demonstrated a rapid resolution of lesions [5]. These two studies demonstrate pharmacokinetics/pharmacodynamics data are sufficient to predict clinical effectiveness.

Finally, the authors seem to have an impression that the regulatory process was abbreviated/expedited. ARS has conducted more than 20 studies with > 750 participants, over 8-years to address all of the FDA’s requests and optimize neffy® dosing. Patients often fail to fill prescriptions or use injectable epinephrine in a timely manner due to a variety of reasons including and especially a fear of needles. It is hoped that neffy® will overcome this patients’ hesitation offering a needle-free safe and effective therapeutic option for the management of anaphylaxis.
